# Are We What We Breathe? Rethinking Air as a Nutritional Source

**DOI:** 10.1016/j.advnut.2024.100360

**Published:** 2024-12-15

**Authors:** Jean Debédat

**Affiliations:** 1 Department of Surgery, University of California Davis School of Medicine, Sacramento, CA, United States; 2 Center for Alimentary and Metabolic Science, University of California Davis School of Medicine, Sacramento, CA, United States

## Introduction

In nutrition research, the gastrointestinal tract has historically been viewed as the sole contributor to nutrient absorption, with the air we breathe viewed as little more than an oxygen provider. The recent review paper “*A Breath of Fresh Air: Perspectives on Inhaled Nutrients and Bacteria to Improve Human Health*” ought to challenge this narrow view, proposing that the air we breathe might also be a source of nutrients and microbes with potential health benefits [1]. In the field of nutrition, few ideas have prompted such a radical rethinking of basic principles than the concept of nutrients and microbes delivered through the air.

## Nutrition Beyond the Plate?

The respiratory tract is uniquely positioned to interact with the environment, which explains why it has been extensively studied as a route for pathogens and diseases. Nevertheless, what makes it perfect for air exchange—its direct exposure to the “outside world,” extensive surface area, high air exchange rate, dense vascularization, and active immune system—also makes it extremely well-suited for nutrient and microbe absorption. This is the direction taken by the authors Dr. Flávia Fayet-Moore and Prof. Stephen R. Robinson, who devised two new concepts: aeronutrients and aeromicrobes. The authors suggest that these airborne elements are absorbed through nasal and pulmonary pathways, where they can influence both local and systemic health. Aeronutrients—airborne vitamins, trace minerals, and fatty acids—are proposed to contribute to dietary intake while aeromicrobes, or airborne bacteria, may play a role in maintaining and diversifying the respiratory and gut microbiomes. This perspective redefines the respiratory tract as a site for nutrient absorption and microbial interaction and further emphasizes how intertwined the respiratory and gastrointestinal tracts are [2].

## The Air as a Nutrient Source—Social Implications

Viewing air as a nutrient source might also shift how we consider our environment and its relationship to our health. Urbanization and industrialization have significantly altered air quality [3], and while the public discourse on air quality often focuses on harmful pollutants [4], the absence of beneficial components in urban air might be as equally concerning.

The authors propose that restoring biodiversity in urban air through initiatives such as urban greening could reintroduce health-promoting aeromicrobes and aeronutrients. Policies promoting green spaces and reducing pollutant exposure could enhance the aerobiome, particularly for marginalized populations with limited access to natural environments [5]. For rural populations, the challenge lies in maintaining the benefits of greater biodiversity while reducing the risks associated with farming activities [6], highlighting the importance of social context in the interplay of environmental exposure and health. Investigating how geography, lifestyle, and socioeconomic factors influence exposure to aeronutrients and aeromicrobes could guide targeted interventions.

These new concepts of aeronutrients and aeromicrobes pave the way toward innovative public health strategies. Could air quality improvements complement dietary guidelines? Could fostering a diverse aerobiome protect against respiratory diseases and improve overall health? The authors argue that these questions related to the air we breathe are crucial, particularly considering the lessons learned during the COVID-19 pandemic [7,8]. Vulnerable populations, such as the immunocompromised and elderly, may benefit the most from interventions or policies that enhance air quality.

## Future Directions

The implications of aeronutrients and aeromicrobes extend into very diverse fields well beyond nutrition. For example, for astronauts exposed to long durations of artificial air, understanding the role and potential benefits of aeronutrients could inform life-support system design to ensure the best health outcomes, especially as time spent in space might increase in the near future. Similarly, in densely populated urban areas, fostering access to biodiverse and cleaner air and improved indoor air quality could mitigate the health disparities caused by environmental degradation.

The authors also suggest that studying the interaction between aeronutrients and the gut–lung axis may reveal new pathways for nutritional interventions. Animal studies have already demonstrated that exposure to diverse aerobiomes can influence microbiota composition and behavior, underscoring the potential for bioactive air components to affect both mental and physical health.

Future research should explore the mechanisms by which aeronutrients and aeromicrobes interact with the human body and their long-term effects on health. Furthermore, this conceptual field requires a robust research framework to emerge. How do we define “good air,” in terms of aeronutrients and aeromicrobes? Experimental standards, metrics, and proper measuring tools defining these emerging concepts will be crucial for integrating these concepts into health care practices and public health policies.

## Conclusion

Air has always been essential for life, but its role in human health may extend far beyond oxygen. By positioning air as a source of nutrients and microbial interactions, this work challenges conventional views and paves the way for a more comprehensive approach to physiology and health. The authors’ insights into aeronutrients and aeromicrobes invite a re-evaluation of how we approach nutrition science, design urban spaces, and craft public health policies. As research advances, these concepts may revolutionize our understanding of the connections between our environment, our physiology, and our health.

## Funding

The author reported no funding received for this study.

## Conflict of interest

The author reports no conflicts of interest.
